# Recent Developments in Metallic Nanomaterials for Cancer Therapy, Diagnosing and Imaging Applications

**DOI:** 10.3390/pharmaceutics14020435

**Published:** 2022-02-17

**Authors:** Dan Nicolae Păduraru, Daniel Ion, Adelina-Gabriela Niculescu, Florentina Mușat, Octavian Andronic, Alexandru Mihai Grumezescu, Alexandra Bolocan

**Affiliations:** 1 Carol Davila University of Medicine and Pharmacy, 050474 Bucharest, Romania; dan.paduraru@umfcd.ro (D.N.P.); daniel.ion@umfcd.ro (D.I.); florentina.musat@drd.umfcd.ro (F.M.); octavian.andronic@umfcd.ro (O.A.); alexandra.bolocan@umfcd.ro (A.B.); 2 Emergency University Hospital of Bucharest, 050098 Bucharest, Romania; 3 Department of Science and Engineering of Oxide Materials and Nanomaterials, Faculty of Applied Chemistry and Materials Science, Politehnica University of Bucharest, 011061 Bucharest, Romania; adelina.niculescu@upb.ro; 4 Research Institute of the University of Bucharest—ICUB, University of Bucharest, 050657 Bucharest, Romania; 5 Academy of Romanian Scientists, Ilfov No. 3, 50044 Bucharest, Romania

**Keywords:** metal-based nanoparticles, nanomedicine, metallic anticancer agents, cancer therapy, drug delivery, hyperthermia, radiotherapy, phototherapy, combined cancer therapies

## Abstract

Cancer continues to represent a global health concern, imposing an ongoing need to research for better treatment alternatives. In this context, nanomedicine seems to be the solution to existing problems, bringing unprecedented results in various biomedical applications, including cancer therapy, diagnosing, and imaging. As numerous studies have uncovered the advantageous properties of various nanoscale metals, this review aims to present metal-based nanoparticles that are most frequently employed for cancer applications. This paper follows the description of relevant nanoparticles made of metals, metal derivatives, hybrids, and alloys, further discussing in more detail their potential applications in cancer management, ranging from the delivery of chemotherapeutics, vaccines, and genes to ablative hyperthermia therapies and theranostic platforms.

## 1. Introduction

Cancer comprises a complex array of diseases that represent one-third of the leading causes of morbidity and mortality worldwide. As traditional therapeutic approaches (i.e., chemotherapy, radiotherapy, and surgery) may result in severe adverse effects or/and unsatisfactory treatment outcomes, intense research has been shifted to integrating nanotechnology in cancer management [1,2,3,4].

Nanomedicine, the overlapping field of nanotechnology and medicine, brings a series of advantages over conventional cancer therapeutics, including multifunctionality, efficient drug delivery, and controlled release of chemotherapeutic agents. These benefits are possible due to the unique physical and chemical properties of nanoparticles (NPs), such as small size, chemical composition, large surface area, tailored shape, and morphology [5,6,7,8,9].

Whether based on polymeric, liposomal, or metallic formulations, NPs naturally traffic to the spleen and lymph organs, being good candidates for delivering immunotherapeutic agents [8]. Moreover, nanomaterials can be used as cytotoxics and/or enhancers of standard chemotherapies, diminishing the side effects associated with conventional drugs, extending their blood circulation time, and preventing drug degradation before reaching the target site [1,10].

Metal-based NPs are particularly appealing in nanomedicine due to their relatively narrow size and shape distribution, long activity period, dense surface functionalization, and capability for optical or heat-based therapeutic strategies. Compared to nonmetallic NPs of similar sizes, the higher density of metallic NPs allows them to be more readily taken up by cells, thus proving advantageous for cancer management strategies [8,11]. In addition, metal NPs were reported to offer better targeting, gene silencing, and drug delivery, especially when functionalized with targeting ligands that provide controlled deposition into tumor cells [6].

**Figure 1:**
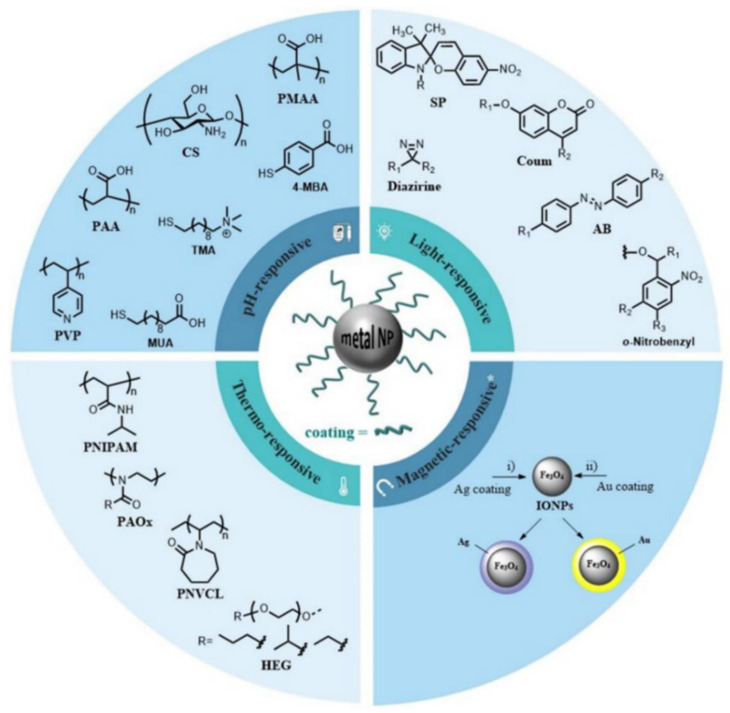
Examples of commonly used moieties for coating metal NPs to produce stimuli-sensitive nanosystems. Reproduced from [12].

Metallic nanoconstructs can remodel the tumor microenvironment (TME) by turning unfavorable conditions into therapeutically accessible ones. For instance, external stimuli (e.g., light, heat, ultrasonic radiation, and magnetic fields) can enhance the targeting ability of metallic NPs towards altering the redox potential of biological systems and generating reactive oxygen species (ROS) that further sensitize target tissues [12]. Furthermore, certain metallic NPs can induce oxidative stress in cancer cells even in the absence of external stimulation [13,14]; internal conditions specific to tumor tissues, such as pH, redox potential, and hypoxia, represent additional viable stimuli for triggering metal-based NPs activity and drug release, enhancing therapeutic efficacy. Furthermore, surface functionalization of metallic NPs with different organic molecules, macromolecules, or noble metal coatings is considered an excellent tool for stabilizing NPs and manipulating their properties towards responding to the above-mentioned stimuli (Figure 1) [12].

Given these advantageous properties, numerous studies have investigated various metal-based nanoparticles as an innovative technology for fighting cancer. NPs made of metals (e.g., gold, silver, iron, zinc, titanium, cerium, and platinum), metal derivatives, metal alloys, metal hybrids, and combinations of metals with other nanomaterials have been increasingly reported in the specialty literature.

In this respect, the present paper aims to thoroughly review metallic nanomaterials that present attractive features for treating malignant diseases and present the possible applications of metal-based NPs in various therapeutic, diagnosis, and imaging approaches, focusing on the newest developments in the field.

## 2. Metallic Nanomaterials

### 2.1. Gold NPs

**Figure 2:**
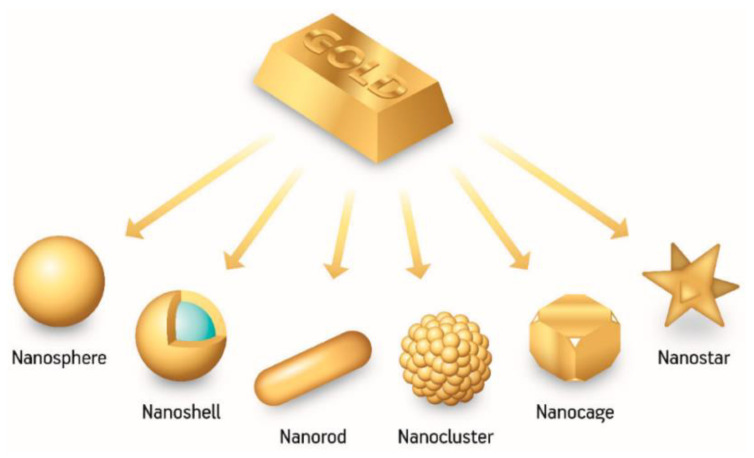
Visual representation of the most common Au NPs assemblies and morphologies in nanomedicine. Reproduced from [18].

Gold nanoparticles (Au NPs) represent one of the most investigated metal-based NPs in medicine [15]. The appealing features of Au NPs count low toxicity and immunogenicity, good biocompatibility, excellent stability, enhanced permeability and retention, inherent immune activation properties, and easily modifiable surface [1,8,11,16]. Moreover, the developments achieved in various chemical and biological synthesis methods allowed the fabrication of Au NPs with tailored sizes, shapes, and structures (Figure 2), endowing them with desired properties. For instance, nanospheres’ small surface area is particularly advantageous for creating efficient cytotoxic agents, and nanocages and nanoshells’ inner cavities are appealing for drug encapsulation, while the large surface available to interact with light per unit of volume of nanorods recommends them for phototherapies. Thus, the versatility and tunability of Au NPs render them suitable for creating excellent delivery vehicles that can achieve targeting and selectivity against cancer cells even without additional molecules [1,17,18,19,20].

Au NPs have also been reported to have tunable optical properties, surface plasmon resonance (SPR), photothermal properties, and surface-enhanced Raman scattering (SERS), being useful tools in phototherapy and photoimaging [16]. SPR is particularly important as this optical property allows Au NPs to be used in near-infrared (NIR)-resonant biomedical modalities, including magnetic resonance imaging (MRI), photoacoustic imaging (PAI), fluorescence imaging, and X-ray scatter imaging [21]. Moreover, Au NPs are of good use in cancer hyperthermia therapy. They can generate heat due to the contained mobile carriers that can be resonant at specific frequencies, depending on the structure of nanoplatforms. The heating effect increases under a plasmon resonance frequency, when all mobile carriers present on the particle resonate [22]. Other applications of Au NPs in cancer management include but are not limited to gene silencing, radiotherapy, and positron emission tomography (PET) imaging [6,21,23].

Additionally, green-synthesized Au NPs were demonstrated to have intrinsic antitumor properties [24], showing promising results when tested against several human cancer cell lines, counting liver cancer [25,26], lung cancer [27,28,29], colon cancer [30,31,32], pancreatic cancer [33,34], breast cancer [35,36], cervix carcinoma [37,38], and ovarian adenocarcinoma [39]. In what concerns the mechanisms of action, the particles were reported to enhance ROS production, change the mitochondrial membrane potential, inhibit the migration assay, activate caspase expression, and downregulate antiapoptotic protein expression, eventually resulting in antiproliferative effects and cancer cells apoptosis.

### 2.2. Silver NPs

Silver nanoparticles (Ag NPs) are another highly investigated material in nanomedicine. Ag NPs have been extensively explored due to their physicochemical and biological properties, including biocompatibility, large surface-to-volume ratio, potent antimicrobial activity, excellent SPR, ease of functionalization, and cytotoxicity against cancer cells [20,40].

Ag NPs have attracted increasing interest in the oncological domain, possessing intrinsic anticancer activity and being demonstrated as effective antitumor drug delivery systems [41,42]. Research has also proven that Ag NPs can modulate the autophagy of cancer cells, either acting as cytotoxic agents themselves, in combination with transported molecules, or in association with other treatments [1].

**Figure 3:**
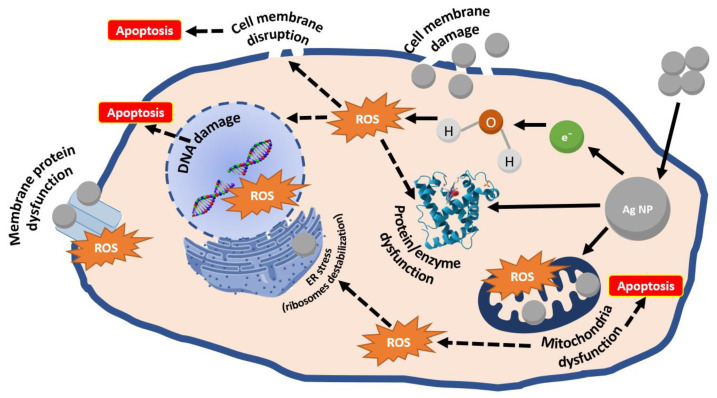
Schematic representation of Ag NPs anticancer mechanisms of action. Created based on information from [44,49,50].

Concerning the mechanisms of anticancer action, Ag NPs were noted to affect membrane fluidity, resulting in facile entry and accumulation in cancer cells, thus causing cancer cells death or hindering their uncontrolled proliferation. Ag NPs can also act by regulating signaling pathways, inducing early apoptosis in the absence of the p53 tumor suppressor [6,40,43]. Moreover, NPs can release Ag^+^ cations that capture electrons, increase intracellular oxidative stress, increase ROS production, reduce ATP levels of cancer cells, and decrease cell proliferation rates [6,44]. Ag^+^ ions are reportedly released mainly in mitochondria and secondarily in the nuclei; there, they interact with DNA, resulting in its fragmentation and resulting in cell death [43,45]. The described Ag NPs mechanisms of action are visually represented in Figure 3; similar paths have also been proposed for other noble metal-based nanoparticles, including Au NPs [13,46], platinum NPs [47], and palladium NPs [48].

Significant results concerning Ag NPs antitumor activity were reported against several human cancer cell lines [43] (Table 1), including hepatocellular carcinoma [51,52,53], breast cancer [54,55,56,57], ovarian cancer [58,59], prostate cancer [60,61,62], colon cancer [30,63,64,65,66], lung cancer [58,60,67,68], and osteosarcoma [69,70].

### 2.3. Iron Oxide NPs

Different types of iron oxides found in nature have started to be explored for synthesizing magnetic NPs, such as magnetite (Fe_3_O_4_), hematite (α-Fe_2_O_3_), and maghemite (γ-Fe_2_O_3_ and β-Fe_2_O_3_) [3,71,72]. Iron oxide nanoparticles (IONPs) exhibit many advantageous properties for biomedical applications, including non-toxicity, biocompatibility, superparamagnetism, chemical inertness, and easily tunable surface [71,73,74].

Regarding cancer care, IONPs have been FDA-approved for clinical testing in cancer diagnosis, imaging, and magnetic hyperthermia therapy, also demonstrating potential in preclinical settings for photothermal and photodynamic therapies [75,76]. One particular ferrofluid formulation developed by MagForce AG has received approval for the treatment of brain tumors, their “NanoTherm” therapy being certified for use on patients from member states of the European Union [77]. IONPs have also been extensively researched for imaging applications. Iron oxide-based particles were noted to be promising contrast agents for different imaging modalities, counting MRI [78,79], fluorescence imaging [80], single-photon emission computed tomography (SPECT) [81], and multimodal imaging [82].

**Figure 4:**
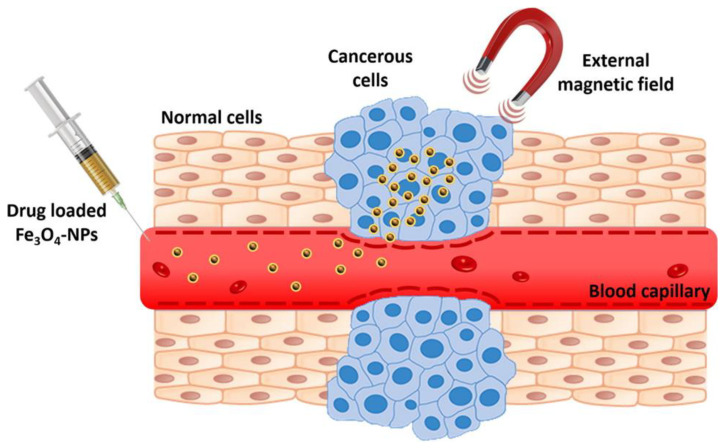
Schematic representation of targeted drug delivery using iron oxide NPs. Reproduced from [3], Elsevier B.V., 2020.

Moreover, IONPs have attracted particular interest in developing magnetic nanoparticle-based drug delivery systems. This is particularly because drug-loaded IONPs have strong targeting ability under external magnetic guidance (Figure 4). Specifically, by applying an external magnetic field, injected IONP-based delivery systems move through blood capillaries towards the desired site, releasing the drug in tumor cells, thus increasing therapeutic efficacy without damaging the neighboring normal cells [3,83]. In addition, their magnetic properties allow the transformation of radiant energy into heat or ROS after applying the local external magnetic field, reducing the adverse effects of cancer therapy [6].

Various such IONPs-based nanosystems have been tested in vitro and in vivo, being reported effective against several types of cancer, including breast cancer [84,85], lung cancer [86,87], liver cancer [88], gastric cancer [89], colorectal cancer [90], prostate cancer [91], and ovarian cancer [92]. The particles showed promising results either alone, in combination with other nanomaterials (e.g., copper, chitosan, aminosilane, and polyethylene glycol) or as carriers of different chemotherapeutic agents (e.g., doxorubicin, docetaxel, and curcumin).

### 2.4. Other Metal-Based Nanomaterials

In addition to the above-discussed NPs, several other metals and metal derivatives started to be investigated in their nanoform as potential candidates for applications in cancer management. For instance, zinc oxide nanoparticles (ZnO NPs) have recently become used in biomedical and cancer applications due to their favorable chemical properties [1]. ZnO NPs exhibit good biocompatibility, antimicrobial, and anticancer activities, gaining increasing popularity in nanomedicine [93,94]. Moreover, ZnO NPs were reported to behave similarly to genotoxic drugs due to their ability to form micronucleus into the cells. This effect was noted to be maximum on glioblastoma multiforma tumor cells, medium on epithelial carcinoma cells, and absent on normal cells [6]. Anticancer activity was also reported for green-synthesized ZnO NPs against several other human cancer cell lines, including colon cancer [95,96], cervical cancer [96], breast cancer [96], lung cancer [97,98], laryngeal cancer [99], and osteosarcoma [100].

Cuprous and copper oxide NPs are other nanomaterials with potential applications in the biomedical field [101]. In particular, plant-synthesized NPs showed pharmacological effects in tumor therapy, such as inducing apoptosis, increasing ROS generation, inhibiting metastasis, and stimulating autophagic cell death [1,6] in colon cancer [102,103], esophageal cancer [104], lung cancer [105], breast cancer [106], cervical cancer [107], renal cell carcinoma [108], and melanoma [109].

Titanium dioxide NPs (TiO_2_ NPs) represent a useful material for photodynamic therapy. Its mechanism of action is based on the excitation of hydrophobic molecules with electromagnetic radiation in the range of visible or UV light towards ROS generation and further induction of apoptosis [6]. Moreover, TiO_2_ NPs were reported cytotoxic in several human cancer cell lines, such as colon cancer [110], breast cancer [111], and osteosarcoma [112].

Cerium oxide NPs have attracted interest in cancer management, especially in radiation therapy and drug delivery of chemotherapeutics. These NPs exhibit the smart capacity of inducing tumor cell death while leaving the surrounding healthy tissues unharmed by radiation and oxidative stress [6]. In this respect, recent studies investigated cerium oxide NPs anticancer potential for treating colon cancer [113,114], pancreatic cancer [115], breast cancer [116,117], and ovarian cancer [114].

Recent studies revealed that bio-synthesized palladium nanoparticles (Pd NPs) present antioxidant, anticancer, antimicrobial, antiproliferative, and photothermal activities while being biocompatible and less toxic than their chemically-synthesized counterparts [118]. Thus, they hold great promise in developing novel and improved cancer therapies, being already evaluated against several cancer cell lines (e.g., lung cancer [48], breast cancer [119], ovarian cancer [120], cervical cancer [121], and colorectal adenocarcinoma [121]).

Advantageous properties have also been reported for platinum nanoparticles (Pt NPs), resulting in increasing interest for applications in biotechnology, nanomedicine, and pharmacology fields. Specifically, Pt NPs exhibit potent antimicrobial, antioxidant, and anticancer activities; SPR; and photothermal properties, which are all highly valuable characteristics for designing performant nanotherapeutics, drug-delivery systems, and bioimaging agents [122,123,124].

### 2.5. Hybrid Metal NPs and Metallic Alloy NPs

**Figure 5:**
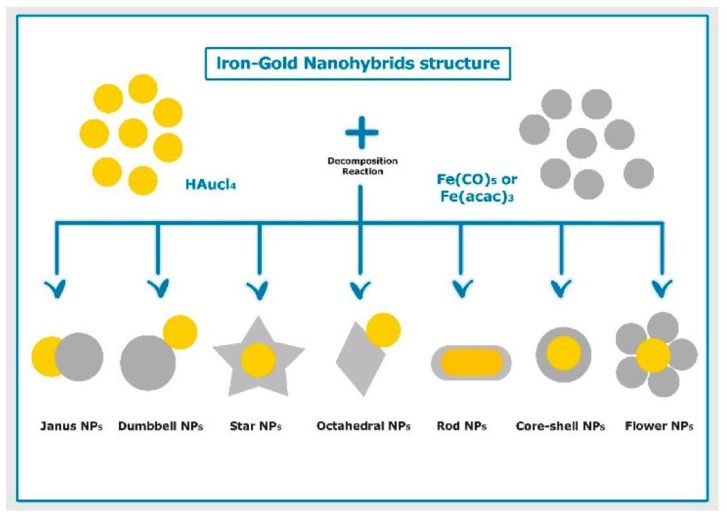
Schematic illustration of different structures of iron–gold hybrid nanoparticles prepared using thermal decomposition method. Reproduced from [125].

In addition to their remarkable individual potential, metals can also be manufactured into hybrid metal NPs towards creating synergistic effects. For instance, bifunctional iron-gold NPs of different shapes, sizes, and structures (Figure 5) are being evaluated for biomedical applications, such as targeted drug delivery, biosensing, photothermal therapy, and immunoassays. The attention these NPs have drawn owes to their beneficial physicochemical properties, including low toxicity, small size, large surface-to-volume ratio, optical characteristics, slow oxidation, increased magnetic susceptibility, and high saturation magnetization [125].

Other hybrid metal-based NPs that are relevant for cancer management include, but are not limited to, MnSe@Bi_2_Se_3_ core-shell nanostructures, FeSe_2_/Bi_2_Se_3_ nanoparticles, Pt@Fe_2_O_3_ nanorods, Au@FeS nanoparticles, Au@Pt nanodendrites, Au@MnO_2_ nanoparticles, and Au@Se nanoparticles [126].

**Figure 6:**
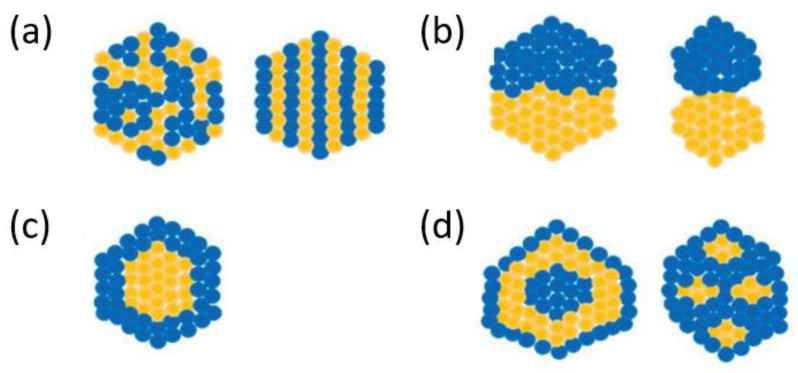
Types of bimetallic alloyed nanoparticles. (**a**) Mixed alloyed nanoparticles; (**b**) sub-cluster segregated alloyed nanoparticles; (**c**) core–shell alloyed nanoparticles; (**d**) multiple core–shell alloyed nanoparticles. Reproduced from [127].

Synergistic effects were also reported when using bimetallic (Figure 6) and trimetallic alloy nanoparticles, which are progressively studied for various applications. Compared to monometallic NPs, alloy NPs benefit from more stable structures and improved properties, possessing superior qualities in biomedical imaging. In particular, iron-based alloy NPs (e.g., Fe-Ni and Fe-Pt) have been employed in MRI as potential contrast agents due to their high magnetic or superparamagnetic property and low toxicity in living cells [127].

## 3. Discussion on Cancer Applications of Metallic Nanomaterials

**Figure 7:**
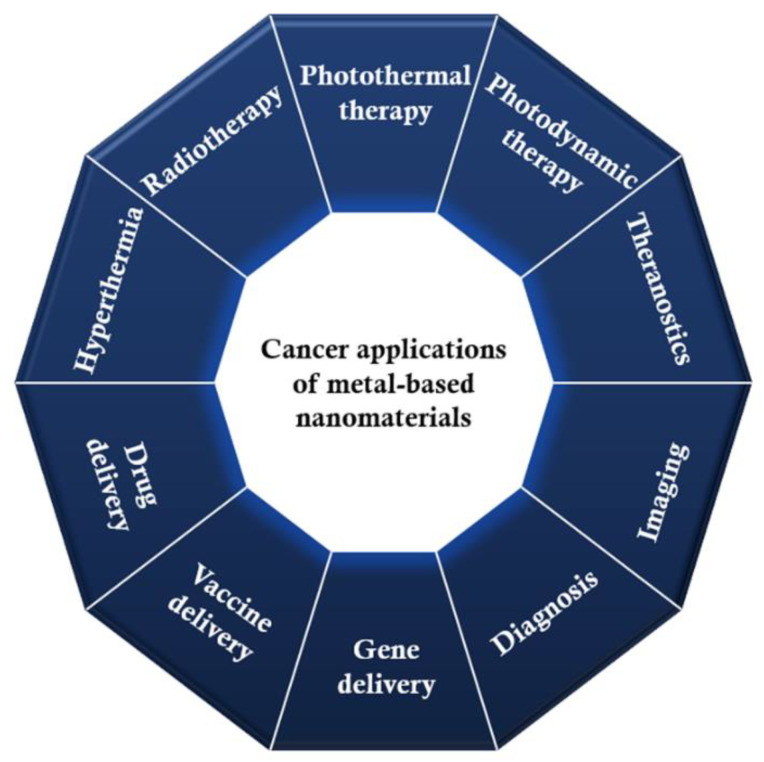
Applications of metal-based nanomaterials in cancer management.

Either alone, as alloys, in various metallic combinations or in association with other nanomaterials, metal-based nanoparticles can be employed in a plethora of applications for better cancer management (Figure 7). In this respect, the following subsections review the role of metallic nanomaterials in detecting and treating cancer, describing some of the most recent advancements in these fields.

### 3.1. Drug Delivery

Metal-based NPs can circumvent the limitations of conventional chemotherapy by providing targeted and controlled release of carried anticancer agents. Thus, carrying drugs via nanoplatforms made of gold, silver, or metal derivatives has become an intense research topic. Studies have reported promising results for a variety of metal-based nanocarriers, either in pristine form, functionalized with targeting moieties or coated with biocompatible layers. Such nanosystems have been proven to enhance in vivo stability, increase drug accumulation in the tumor site, improve the therapeutic effectiveness of carried drugs, and reduce systemic toxicity. Moreover, they can ensure sustained/programmable or on-demand drug release by responding to internal or external stimuli, respectively [128,129,130].

**Figure 8:**
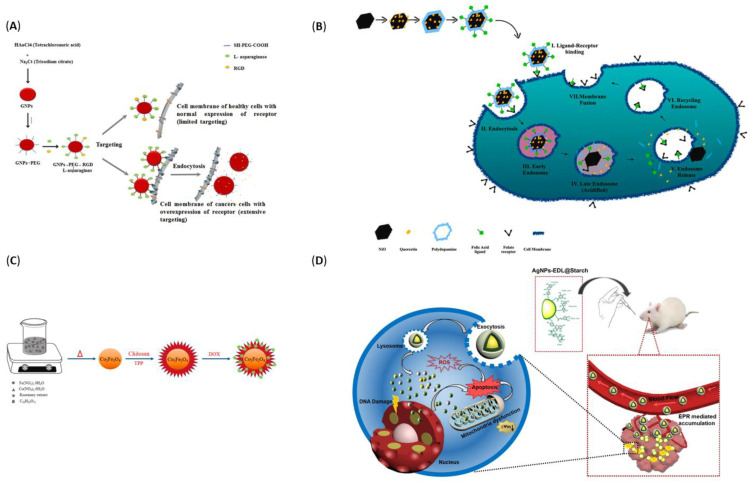
(**A**) Schematic representation of the formation of Gold NPs–PEG-RGD-Asparaginase (conjugate) and cellular uptake. Adapted from [131], Elsevier B.V., 2020. (**B**) Schematic representation of formation of honeycomb structured nickel oxide nanoparticles and intracellular drug release mechanism. Reproduced with permission from [137], Elsevier B.V., 2021. (**C**) Schematic representation of the preparation and final structure of cobalt ferrite NPs for doxorubicin delivery. Reproduced with permission from [139], Elsevier B.V., 2021. (**D**) Schematic representation of EDL-encapsulated AgNPs oral administration and cancer therapy mechanism of AgNPs-EDL@Starch. Reproduced with permission from [133], Elsevier B.V., 2021.

In order to emphasize the versatility of metal-based NPs for drug delivery, Table 2 has gathered several top-recent developments in the field. Moreover, Figure 8 offers a visual perspective over the discussed nanostructures.

**Table 2:** Examples of recently developed metal-based drug delivery systems for cancer therapy.

Material	Morphology	Carried Drug	Properties	Results	Ref.
Gold	PEG-modified nanospheres (with Arg-Gly-Asp (RGD) peptide as targeting agent)	L-asparaginase	▪ Average size: 29.24 ± 5.38 nm	▪ NPs improved drug bioavailability and anticancer activity ▪ Significant antioxidant effects ▪ High tumor-targeting efficacy and distribution in MCF-7 cells ▪ Initiation of apoptosis and promotion of cell cycle arrest at the G2/M ▪ Upregulated pro-apoptotic p53, while downregulating antiapoptotic Bcl-2	[131]
Silver	Nanospheres	Paclitaxel	▪ Average size: ~10 nm	▪ Nontoxic to noncancerous HUVEC cells ▪ More effective than paclitaxel alone in all tested cells (i.e., MDA-MB-231, MCF-7, 4T1, Saos-2) ▪ Saos-2 cells were ~10 times more sensitive to paclitaxel-bonded Ag NPs that to the bare drug	[132]
Silver	Nanospheres (coated with starch)	*Euphorbia dracunculoides* Lam. (EDL) plant extract	▪ Average size: 42.5 ± 1.54 nm ▪ Loading capacity: up to 82.5% ▪ Encapsulation efficiency: up to 85% ▪ Zeta potential: −29.64 ± 0.09 mV	▪ The surface modification increased biocompatibility ▪ pH-triggered drug release ▪ Enhanced antioxidant potential ▪ Accumulation in cancer cells and induction of early and late apoptosis in RAW264.7 and SCC7 cells	[133]
Magnetite	Nanospheres (coated with polyvinyl alcohol-zinc/aluminum-layered double hydroxide)	Sorafenib	▪ Average size: ~95 nm ▪ Saturation magnetization: 57 emu/g ▪ Remanent magnetization: 2.706 emu/g	▪ No cytotoxicity against 3T3 fibroblasts ▪ More potent than bare drug against HepG2 liver cancer cells ▪ The drug was more easily released under an acidic environment	[134]
Magnetite	Nanospheres (surface modified with Pluronic F127 and branched polyethylenimine)	Doxorubicin	▪ Size range: 10–20 nm ▪ Zeta potential: −20.5–4.87 mV ▪ Saturation magnetization: 54.5–65.5 emu/g	▪ pH-/thermo-responsive drug delivery system ▪ Sufficient magnetic strength to allow navigation towards the desired site ▪ Enhanced the therapeutic effect of the drug	[135]
Maghemite	Hollow nanospheres (functionalized with polyethylene glycol)	Doxorubicin	▪ Average hydrodynamic size: ~175 nm ▪ Specific surface area: 266.1 m^2^/g ▪ Saturation magnetization: 16.3 emu/g	▪ Highly sensitive to alternating magnetic field and pH ▪ Precise drug release to desired tissues	[136]
Nickel oxide	Honeycomb-structured nanoparticles (coated with folic acid-decorated polydopamine)	Quercetin	▪ Average size: 35 nm ▪ Average pore volume: 0.312 cm^3^/g ▪ Average pore size: 11.44 nm ▪ Loading capacity: up to 51% ▪ Encapsulation efficiency: 51%	▪ Surface modification increased biocompatibility and reduced hemolysis ▪ Highly controlled drug release in physiological system compared to TME ▪ Strong anticancer activity at very low concentration ▪ Cytotoxic effects against Vero cells and MDA-MB-231 in a dose-dependent manner	[137]
Zinc oxide	Hexagonal shaped nanoparticles	Quercetin	▪ Average size: 21–39 nm	▪ pH-dependent drug-release, with higher releasing rate in acidic medium ▪ Stable under physiological pH, indicating that the nanosystem can be retained in the blood stream up to particular time point without causing considerable side effects ▪ High biocompatibility with 3T3-L1 cells ▪ Effective inhibition of breast cancer cells (MCF-7) growth	[138]
Cobalt ferrite	Polygonal nanoparticles (coated with chitosan)	Doxorubicin	▪ Average size: 38 nm ▪ Saturation magnetization: 50 emu/g ▪ Drug loading: up to ~89%	▪ Excellent biocompatibility ▪ Non-toxic nanosystem ▪ High drug-release at the pH of cancer tissue ▪ Good cell death rates in breast cancer cell line MCF-7 cells	[139]
Copper oxide	Nanospheres (coated with bovine serum albumin)	Methotrexate	▪ Average size: 23.78 ± 1.52 nm ▪ Loading efficiency: 8.70 ± 2.11%	▪ Significant cytotoxicity against MDA-MB-231 cell line ▪ Faster drug release in the presence of proteinase K enzyme	[140]

As it can be observed from Table 2, not only the classic trio of metal-based nanomaterials (i.e., gold, silver, and iron oxide) have resulted in promising drug delivery applications, but also less investigated materials (e.g., cobalt ferrite, cobalt oxide, and nickel oxide) can be successfully employed in cancer therapy. The tested particles showed enhanced anticancer activity compared to bare chemotherapeutic drugs (even up to 10 times for silver NPs in certain cell lines [132]). Furthermore, the harmful potential of the carrier metals and carried drugs towards healthy tissues was reduced by adding biocompatible polymers, proteins, and targeting agents. Moreover, the differences in the biological activity of the presented nanosystems can be explained by their size and shape variability, nanospheres of smaller diameters being preferentially taken up by the target cells compared to their larger counterparts with different morphologies.

**Figure 9:**
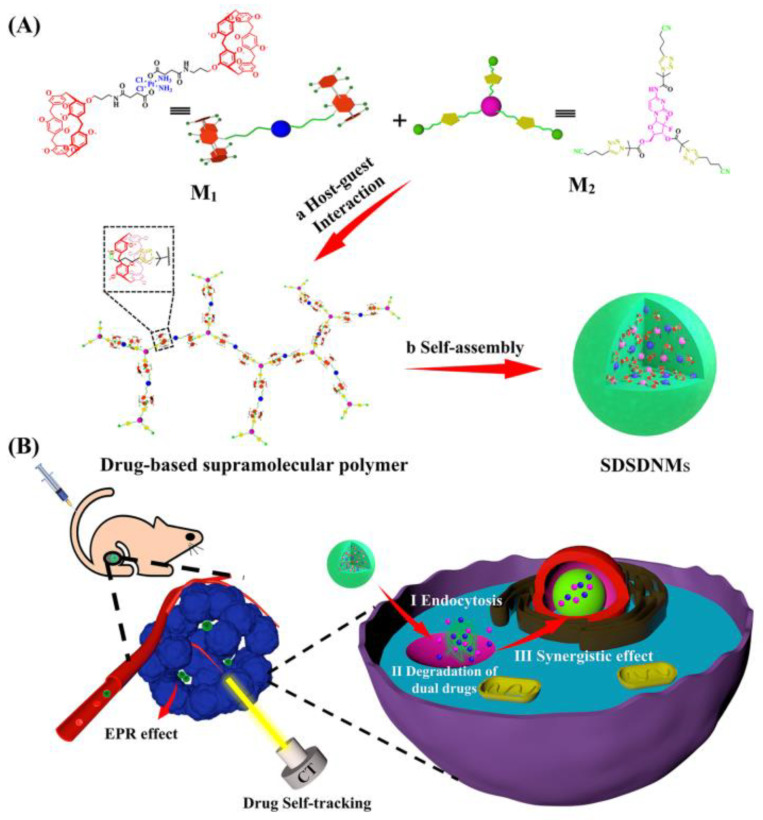
Schematic representation of the SDSDNMs developed by Liu et al. [141]. (**A**) Construction process. (**B**) Accumulation at the tumor site. Reproduced with permission from [141], American Chemical Society, 2021.

Another interesting novel approach is the use of supramolecular drug self-delivery systems (SDSDSs), which comprise active drug-building blocks linked through supramolecular interactions. One metal-based SDSDS is proposed by Liu et al. [141], who have developed platinum-containing supramolecular drug self-delivery nanomicelles (SDSDNMs) able to inhibit tumor growth while preserving good safety towards normal organs (Figure 9). The authors concluded that the newly designed system might provide promising opportunities in the field of synergistic combination chemotherapy.

One more drug delivery strategy gaining interest in recent years is the use of implantable systems for targeted delivery without involving an external magnet. Specifically, implants can be employed as delivery vehicles of drugs, antimicrobial, anti-inflammatory, and immunomodulatory agents to desired sites, including surgery cavities to prevent potential cancer recurrence. Nonetheless, specific requirements must be fulfilled by these implants, counting drug release at a proper concentration and distance from the tumor, biocompatibility, antibacterial activity, and ability to surpass immune system recognition [142]. In this respect, Ge et al. [143] have created an iron oxide/poly(lactic-co-glycolic acid) implant scaffold with high magnetism (up to 40% *w*/*w* magnetic beads). The designed scaffold is biocompatible, durable, and effectively attracts nanodrugs to its surface, achieving targeted delivery without the application of an external magnetic field. Thus, it aids in the accumulation of drugs to tumor cells, improving therapeutic outcomes. This approach holds great promise for developing more precise medical treatments in the future.

### 3.2. Vaccine and Gene Delivery

In addition to the use of chemotherapeutic agents, cancer vaccination represents another effective method for preventing or curing cancers. Cancer vaccines are based on tumor antigens administered as nucleic acids, tumor lysates, full proteins, or short peptides that can induce strong cellular and humoral immunity. Nonetheless, vaccines necessitate adjuvants to reach their maximum efficacy potential, improve the strength and longevity of immune responses, and reduce doses and side effects [144,145,146,147].

In this respect, metalloimmunology has the potential to revitalize cancer vaccines, as nutritional metal ions (e.g., Ca^2+^, K^+^, Fe^2+/3+^, Zn^2+^, and Mn^2+^) play important roles in many biological activities, including key immune processes. Thus, a wide variety of metals can be considered good adjuvants for nanovaccines, enhancing the generated immune responses through transcellular or intracellular signaling cascades against neoplastic transformation. More specifically, metal ions are suitable candidates for ensuring abrupt and timely immune responses, achieving effective immune regulation, and producing fewer toxicity concerns than conventional therapies [144]. Moreover, metallic NPs can be used as antigen delivery vehicles, being able to improve their uptake by dendritic cells (DCs) (or other antigen-presenting cells); this is further reflected in an increased antitumor cytotoxic T cell response [8].

Table 3 comprises several examples of metal-containing formulations for cancer vaccines delivery, highlighting the variety of tested materials.

According to the studies presented in Table 3, it can be concluded that metal-based nanoformulations hold great promise for vaccine development in various forms, including nanospheres, nanocapsules, and nanowires. By delivering immunogens in a controlled manner, metal derivative nanoconstructs were observed to promote the activation of immune cells and significantly inhibit tumor growth. Moreover, in order to enhance the compatibility and stability of the particles in the biological media, researchers chose to coat them with polymer or lipid layers.

Following similar considerations of protecting the freight and enhancing induced cytotoxicity, metal nanoparticles can be used as carriers of negatively charged nucleotides (DNA and RNA), particularly due to their high positive surface charge. One specific phenomenon for which metal-based NPs hold great promise is gene silencing. They can carry antisense nucleotides that downregulate specific gene expression in tumor cells. As the strength and duration of the silencing response depends on the amount of siRNA delivered to the target site, metal NPs protective capacity can be enhanced by lipid or polymer coatings. Consequently, the load is not affected by RNase, increasing the half-life of siRNA and decreasing the required dose [6].

### 3.3. Magnetic Hyperthermia

A valuable tool in cancer management that uses the unique properties of metallic NPs is ablative hyperthermia [72]. This general term covers therapeutic approaches in which applied energy is transformed into heat by certain biocompatible metal-based nanomaterials. Increasing the temperature of tumor tissues kills cancer cells or sensitizes them to radiation or chemotherapeutic agents. Moreover, these therapeutic approaches increase blood flow in tumors, induce cytotoxicity, and disrupt tumor vasculature, further releasing tumor-specific antigens and danger signals that alert the immune systems. Thus, despite being only locally applied, ablative therapies may result in systemic immunity, exhibiting abscopal effects. In addition, ablative hyperthermia can be produced by external stimuli, including local external magnetic field (magnetic hyperthermia), radiofrequency (radiotherapy), and light of specific wavelength (photothermal therapy) [1,8,155,156]. Each of these therapies is further discussed in distinct consecutive sections.

**Figure 10:**
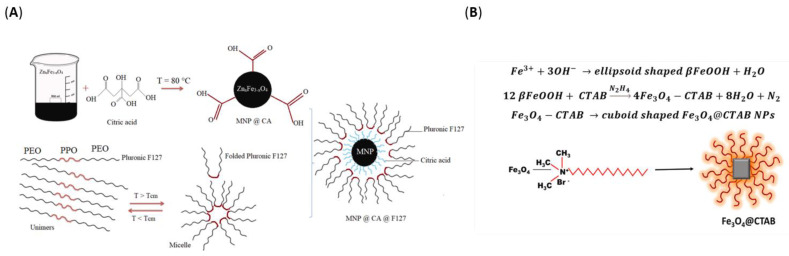
(**A**) Schematic representation of the surface modification of the magnetic nanoparticles with citric acid (CA) and pluronic F127. Reproduced with permission from [159], Elsevier B.V., 2022. (**B**) Schematic representation of the formation mechanism of Fe_3_O_4_@CTAB nanocuboids. Reprinted with permission from [162], Elsevier B.V., 2021.

Magnetic hyperthermia is the noninvasive technique in which an alternate magnetic field (AMF) remotely induces local heating through magnetic energy losses of magnetic nanoparticles. By ensuring precise uptake of magnetic NPs by tumor cells, the adverse effects in surrounding normal tissues can be significantly reduced. Generally, iron oxide NPs can be delivered intratumorally and heated at 41–50 °C under an AMF [1,71]. Nonetheless, other iron-containing nanoconstructs have been recently developed for application in cancer magnetic hyperthermia (Table 4, Figure 10).

**Table 4:** Examples of recently developed metal-based nanosystems for application in cancer magnetic hyperthermia.

Material	Morphology	Properties	Ref.
Carbothermal treated iron oxide	Nanospheres with oxygen vacancies	▪ Size range: 5.1–225.6 nm ▪ Saturation magnetization: 5.8–31.3 emu/g ▪ Specific absorption rate: up to 71.6 W/g	[157]
Magnetite	Nanospheres (coated with dextran)	▪ Average size: 10 nm ▪ Saturation magnetization: 40–60 emu/g ▪ Specific power absorption: 132 W/g	[158]
Zn-substituted magnetite	Irregular hexagonal nanoparticles (coated with citric acid and pluronic F127)	▪ Mean hydrodynamic size: 436–626 nm ▪ Specific loss power: up to 539 W/g ▪ Intrinsic loss power: up to 7.26 nHm^2^kg^−1^	[159]
Gd-doped maghemite	Nanoparticles of almost spherical shape along with some aggregation	▪ Size range: 8.73–11.06 nm ▪ Saturation magnetization: 39.35–52.13 emu/g ▪ Specific absorption rate: up to 140 W/g	[160]
Silver-iron oxide composite	Irregular-shaped particles agglomerated to some extent	▪ Size range: 2–24 nm ▪ Specific loss power: up to 43 W/g ▪ Intrinsic loss power: up to 0.81 nHm^2^kg^−1^	[161]
Iron oxide	Cuboidal-shaped nanoparticles (functionalized with CTAB)	▪ Average edge length: ~80 nm ▪ Saturation magnetization: 71 emu/g ▪ Specific loss power: up to ~1036 W/g	[162]
Cobalt ferrite	Nanospheres (coated with chitosan)	▪ Average size: 13 nm ▪ Saturation magnetization: ~62 emu/g ▪ Specific absorption rate: up to 105 W/g	[163]
Copper ferrite	Mesoporous spherical structures	▪ Hydrodynamic size: ~91.2 nm ▪ Saturation magnetization: ~32.7 emu/g ▪ Specific absorption rate: up to ~192 ± 7 W/g	[164]
Copper ferrite	Pseudo-cubical shaped particles	▪ Hydrodynamic size: ~25.6 nm ▪ Saturation magnetization: ~24.5 emu/g ▪ Specific absorption rate: up to ~116 ± 6 W/g	[164]
Manganese ferrite	Uniform nanospheres with some agglomeration	▪ Average size: ~25 nm ▪ Saturation magnetization: 54.18–59.67 emu/g ▪ Specific absorption rate: 217.62 W/g	[165]

### 3.4. Radiotherapy

The inherent optical, electrical, and conductive properties of metal-based nanoparticles have rendered them appealing for use in radiotherapy. Metal NPs can increase the specificity of radiations to the desired site in such a manner that the maximum dose is delivered to the tumor tissue, while toxicity and damage are avoided in healthy tissues. Metal-based NPs were reported to increase intracellular ROS production from the ionizing radiations, increase oxidative stress levels in tumor cells, increase apoptosis rates, and reduce clonogenic survival [5,6].

A variety of metals and metal derivatives can be used in radiotherapy, including gold, silver, platinum, titanium oxide, zinc oxide, and more. Metallic materials with atomic number between 22 and 83 have been researched in radiation therapy for a broad range of purposes, such as radiation dose enhancement, hyperthermia induction, controlled drug delivery, and theranostic applications [166]. In particular, the high atomic number and mass-energy coefficient of Au NPs and Ag NPs make them suitable candidates for radiosensitization in cancer imaging and therapy, while the unique physicochemical properties and high X-ray absorption efficiency of platinum-based or hafnium-based NPs recommend these nanoplatforms as ideal radiosensitizers [6,167].

Despite the physical concepts, the role of biological mechanisms has also started being investigated in recent years for radiosensitization purposes. Specifically, gold nanoparticles have been investigated whether they could influence cell response to radiation by five “R” factors (i.e., repair, redistribution, repopulation, reoxygenation, and intrinsic radiosensitivity) [46]. For instance, it has been reported that the presence of Au NPs produces a downregulation of thymidylate synthase, which is essential for DNA damage repair in the radioresistant S-phase cells [168]. Au NPs radiosensitisation ability has also been associated to a decrease in thioredoxin reductase (one of the main redox reactions regulators) activity, weakening the detoxification system [169].

**Figure 11:**
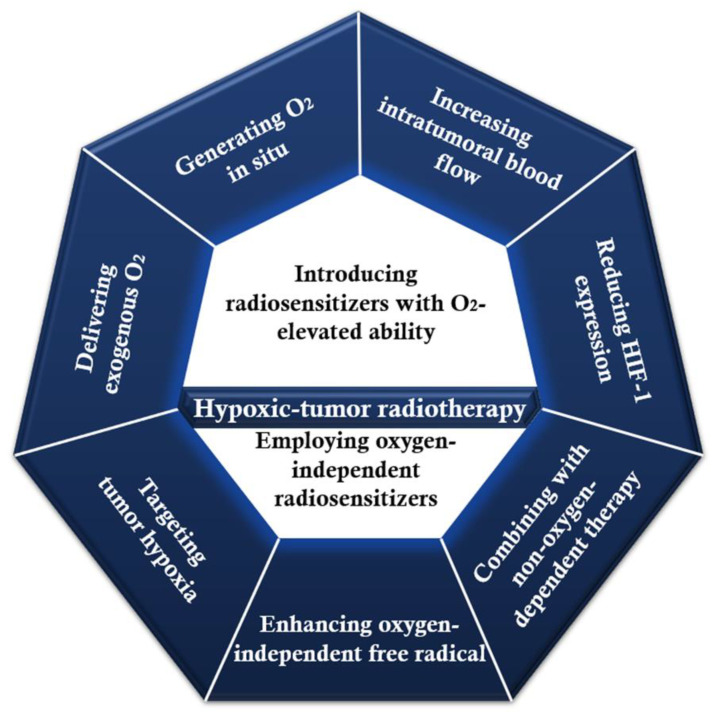
Potential strategies employing metal-based NPs for hypoxic-tumor radiotherapy. Created based on information from [170].

Tumor tissues generally lack a proper amount of oxygen; thus, special strategies (Figure 11) must be considered for hypoxic conditions, as they contribute to chemoresistance and metastasis [142,170]. One interesting example is proposed by Chen et al. [171], who have prepared folic acid-modified enzyme-like hafnium-based manganoporphyrin metal–organic framework nanoparticles (MnTCPP–Hf–FA MOF NPs). The overall goal of the nanosystem was to overcome hypoxia-induced radioresistance and prevent a postoperative recurrence. Hf was chosen particularly due to its high atomic number and ability to absorb X-ray energy and convert O_2_ and H_2_O into ROS, while the MnTCPP ligand was included owing to its enzyme-like ability to decompose endogenous H_2_O_2_ into O_2_ for enhancing radiotherapy in hypoxic conditions. The as-designed MOF NPs were reported to effectively inhibit melanoma growth and prevent recurrence with only one X-ray irradiation after intravenous injection. Therefore, the nanosystem has great potential for overcoming the radioresistance challenge of hypoxic tumors.

### 3.5. Phototherapy

Light has been long used for its healing potential in the treatment of various diseases. In the context of cancer treatment, two major therapeutic alternatives exploit light, namely photothermal therapy (PTT) and photodynamic therapy (PDT) [172]. These phototherapies attracted attention for various combinations with light-responsive or light-triggered NPs towards enhancing treatment efficiency and specificity [173].

PTT can be considered a form of ablative hyperthermia in which photothermal agents (PTAs) are used for the conversion of light into heat, resulting in the selective death of cancer cells under laser application [17]. More specifically, radiative excitation excites PTA, moving it to a higher level and resulting in thermal vibration emissions that kill tumor cells [174]. Therefore, tight control over temperature and temperature gradient is mandatory in order to avoid damaging surrounding normal tissues. Consequently, research started to focus on designing new nanostructures with optimized thermoplasmonic properties [22]. Particular attention has been drawn to Au NPs due to their SPR properties resulting from energy excitation achieved by irradiating them with specific wavelength light [16].

Nonetheless, recent studies also directed their investigations towards designing and evaluating PTAs made of other less-explored metal-based nanomaterials. For instance, Sharker and colleagues [175] reported the preparation of biocompatible tungsten oxide NPs functionalized with dopamine-conjugated hyaluronic acid. Under in vitro NIR irradiation, the particles displayed a rapid and significant rise in photothermal heat against MDAMB and A549 cancer cell lines, whereas in vivo studies proved long-term biocompatibility and efficient photothermal conversion with time-dependent tumor target accumulation. More recently, Sun et al. [176] developed tunable liquid metal nanoparticles with sphere-to-rod morphologies. Out of the tested shapes and compositions, gallium nanorods exhibited outstanding photothermal conversion efficiency and showed distinct temperature rise compared to gallium nanospheres and gallium-indicum alloy nanorods. These smart nanoliquid metals enhanced PTT in model animals, paving the way for further applications in tumor therapy and imaging. Alternatively, Yang et al. [177] have fabricated heterogeneous NPs with gallium-indicum alloy cores and metal shells (platinum, gold, silver, or copper). Particularly promising results have been obtained for GaIn@Pt NPs as they considerably increased photothermal conversion efficiency and improved thermal stability under NIR irradiation. These nanoconstructs also displayed a good Fenton-like catalytic effect, resulting in the conversion of endogenous tumor H_2_O_2_ into ROS. Moreover, NPs were further optimized by modification with polyethylene glycol, resulting in improved biocompatibility, efficient tumor homing after intravenous injection, and effective NIR-triggered photothermal-chemodynamic synergistic outcomes in a mouse tumor model.

PDT is another promising cancer treatment that can benefit from advancements in the field of metal-based NPs. PDT requires the use of light with specific wavelengths for the activation of photosensitive chemicals (known as photosensitizers PS) towards generation of ROS and further destruction of cancer cells. However, conventional PS may face a poor specific uptake in tumor cells, posing a threat on healthy neighboring tissues. Thus, involving nanotechnology in this type of therapy became fundamental for obtaining maximum results while avoiding side effects [11,16,178].

In order to improve PS accumulation in the target tissue, these substances can be carried and delivered to the site by metal-based NPs. Compared to bare PS, such nanoplatforms exhibit long cycle time, slow degradation, and targeted and controlled release, benefiting also form enhanced permeability and retention effect [172]. Au NPs of many forms (e.g., nanocages, nanorods, nanoshells, and nanoclusters) can enhance PDT performance by sensitizing singlet oxygen formation, generating ROS, providing spatiotemporal control, and diminishing the undesirable effects of clinically used PS [16,179,180,181]. Titanium oxide has also attracted interest in PDT for treating malignant tumors, especially due to its adjustable bandgap, band position, and excellent photostability. Similarly, due to its electronic structure, zinc oxide has been investigated as a photo- or sonosensitizer for cancer therapy, displaying cytotoxic effects when exposed to appropriate external stimuli. Another metal derivative that has been demonstrated to be effective in PDT is manganese oxide. Manganese oxide-based nanoconstructs can generate O_2_ in situ by reacting with H_2_O_2_ from TME, while also consuming glutathione. Thus, it represents an interesting delivery vehicle for PSs, especially in hypoxic tumors [172,181,182].

### 3.6. Diagnosis

Early detection and thorough monitoring of cancer evolution are essential processes in controlling and preventing the disease [183]. Thus, despite advancements in various therapies, special focus is also required in developing more performant imaging and diagnosis modalities.

As there is only a low content of cancer biomarkers in the early phase of cancer, ultrasensitive and selective tools are mandatory for their detection. In this context, metal NPs have emerged as convenient solutions due to their unique optoelectronic properties and ease of functionalization. Materials such as gold, silver, and copper represent appealing candidates for developing analytical scaffolds, especially because their SPR bands are in the visible region. However, copper use is limited by its ease of oxidation, most of the studies involving Au and Ag-based nanoconstructs [184].

One innovative example of ultrasensitive surface-enhanced Raman scattering (SERS) immunoassay was recently developed by Yang et al. [183]. The scientists fabricated a core-shell nanostructure of Au@Ag that could detect α-fetoprotein (AFP), a biomarker of liver cancer. The nanoplatform exhibited excellent analytical performance of the SERS immunoassay in the range from 0.5 to 100 pg/mL with a limit of detection of 0.081 pg/mL (3*σ*), demonstrating potential applications in clinical diagnosis.

### 3.7. Imaging

Interesting possibilities also arise from the use of Au NPs in the dual detection of prostate cancer via optical imaging (OI) and positron emission tomography (PET). In this respect, Pretze et al. [23] developed a complex nanosystem consisting of Au NPs decorated with a NIR dye and NODAGA chelator, which were further radio-labeled with ^64^Cu. The as-designed metal-based nanoplatform displayed favorable diagnostic properties concerning detection, biodistribution, and clearance, recommending these constructs for future therapeutic concepts.

Superparamagnetic iron oxide nanoparticles (SPIONs) represent another extensively researched metal derivative extensively studied for imaging applications. More specifically, SPIONs are investigated as contrast agents for visualizing tumors and metastatic cancer in different tissues, including the liver, spleen, and lymph nodes. SPIONs are attractive for such applications as they can reduce the relaxation time of the surrounding protons owing to their superparamagnetic behavior, being particularly suitable candidates for MRI [73].

### 3.8. Theranostics

**Figure 12:**
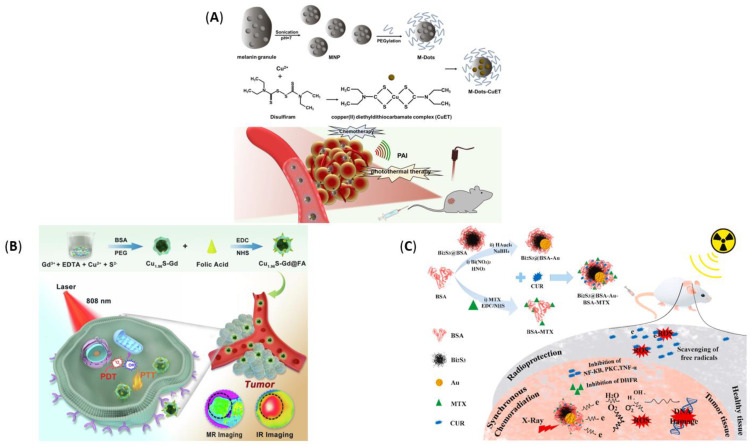
(**A**) Schematic representation of the synthesis of M-Dots-CuET and the process of PAI-guided chemo/photothermal therapy using M-Dots-CuET. Reproduced with permission from [191], Elsevier B.V., 2021. (**B**) Schematic representation of the preparation of dual-modal MR/IR imaging-guided synergistic PTT/PDT with Cu1.96S-Gd@FA nanoparticles. Reproduced with permission from [192], Elsevier B.V., 2022. (**C**) Schematic representation of the synthesis process and tumor ablation mechanism of Bi2S3@BSA-Au-BSA-MTX-CUR. Reproduced from [193], Elsevier B.V., 2022.

In the continuous effort to create better tools for cancer management, multifunctional nanoparticles, called theranostics, emerged as performant alternatives to conventional therapeutics and imaging agents. Thus, numerous studies have recently investigated a myriad of nanomaterials that can encapsulate and co-deliver drugs, imaging moieties and genes, and even detect tumor cells by binding to specific receptors. In order to emphasize the developments encountered for metal-based NPs as theranostics, several examples have been gathered in Table 5 and Figure 12.

**Table 5:** Examples of recently developed metal-based theranostics.

Material	Morphology	Properties	Observations	Ref.
Silver	Quasi-spherical nanoparticles	▪ Average size: <50 nm ▪ Hydrodynamic diameter: ~95 nm ▪ Zeta potential: −14 mV	▪ NPs were biosynthesized using the leaf extract of *Zinnia elegans* ▪ No anticancer drug, targeting moiety, or fluorescent molecule(s) were added to the NPs ▪ Demonstrated anticancer activity in vitro ▪ Illustrated NIR-based bioimaging when intraperitoneally injected in C57BL6/J mice	[128]
Silver	Spherical and rod-like nanoparticles	▪ Average size of NPs obtained with ethylene glycol: 15.58 ± 8.28 nm ▪ Average size of NPs obtained with tetraethylene glycol: 72.44 ± 21.82 nm	▪ NPs were stabilized with polyvinylpyrrolidone ▪ NPs entered cancer cells, exhibiting intense green fluorescence in tested cell lines (MCF-7 and U87-MG) ▪ NPs efficiently internalized in tumor cells through enhanced permeability and retention effect, without causing hemolysis in red blood cells	[185]
Iron oxide	Nanospheres (coated with boiling rice starch extract)	▪ Average size: 86 ± 3.6 nm ▪ Zeta potential: −2.1 mV (at pH 4.5), −4.2 mV (at pH 7.2), and −7.2 mV (at pH 9.0) ▪ Saturation magnetization: ~70.65 emu/g ▪ Drug loading: ~78%	▪ NPs were loaded with doxorubicin ▪ Exhibited excellent photothermal stability, with a high photothermal conversion efficiency ▪ Showed high NIR absorption for photoacoustic imaging-guided PTT ▪ Doxorubicin was preferentially released at acidic environment, specifically targeting cancer cells	[186]
Iron oxide	Nanospheres (coated with porous calcium phosphate)	▪ Size range: 10–20 nm ▪ Relaxivity: 845.71 mM^−1^S^−1^ ▪ Drug loading: 89.6% after 48 h	▪ NPs were loaded with curcumin ▪ Ensured a slow release of the anticancer agent ▪ Strong shortening in the T2 relaxation time ▪ Potential negative contrast agent for MRI	[187]
Iron oxide	Nanospheres (coated with amorphous silica)	▪ Average diameter (bimodal distribution): 70.8 ± 5.8 and 116.8 ± 21.8 nm ▪ Saturation magnetization: 9 emu/g ▪ Drug loading: up to 34% ▪ Specific absorption rate: 24 W/g	▪ NPs were functionalized with curcuminoids ▪ Good colloidal stability, dispersibility and magnetic properties ▪ Suitable for magnetic hyperthermia, fluorescence imaging, and drug delivery	[188]
Gold-iron oxide	Core (Fe_3_O_4_)-shell (Au) structure	▪ Size range: 5–10 nm	▪ NPs induced ROS production ▪ Efficiently internalized into PC3 cells ▪ Exhibited cytotoxicity in cancer cells under X-ray radiations ▪ Dose-dependent anticancer activity, reaching ~95% cell deterioration for a concentration of 20 μg/mL ▪ The specific accumulation of NPs in cancer cells prevented destruction of healthy cells	[189]
Iridium oxide	Sphere-like structure	▪ Average diameter: 30 nm ▪ Hydrodynamic diameter: ~55 nm ▪ Zeta potential: −0.407 mV	▪ NPs were functionalized with split DNAzyme precursor and doxorubicin ▪ Fluorescence imaging studies proved the specificity and feasibility of the NPs ▪ Drug release was photothermally controlled ▪ Excellent synergistic effects against cancer cells under NIR ▪ In vivo studies demonstrated great inhibition of tumor growth	[190]
Copper(II) diethyldithiocarbamate (CuET)	Complex loaded with ultrasmall melanin dots	▪ Average size (of M-dots): ~8 nm ▪ Hydrodynamic diameter (of M-dots): 87.3 ± 3.1 nm ▪ Zeta potential (of the system): 18 mV	▪ Excellent biosafety and biocompatibility ▪ CuET significantly enhanced the water solubility of melanin dots ▪ Good photoacoustic and chemo/photothermal therapy properties ▪ Good tumor accumulation and excellent tumor proliferation inhibition ▪ Combined with PTT, the nanosystem produced a tumor growth inbition of 78.6%	[191]
Copper sulfide	Nanospheres	▪ Average size: 11.8 ± 2.23 nm ▪ Longitudinal relaxivity: up to 12.9 mM^−1^·s^−1^ ▪ Zeta potential: −18.0 ± 3.0 mV	▪ NPs were surface-functionalized with gadolinium and modified with folic acid (FA) ▪ FA enabled NPs targeting, consequently enhancing cellular uptake and therapy efficacy ▪ The system integrates MR/IR dual-modal imaging and PTT/PDT into one nanoplatform ▪ Great potential in anti-breast cancer therapy	[192]
Bismuth sulfide-gold	Nanospheres	▪ Average size: ~8.5 ± 3.0 nm ▪ Hydrodynamic diameter: 152.30 ± 8.90 nm ▪ Zeta potential: −28.50 mV	▪ NPs were conjugated with methotrexate and curcumin ▪ Enhanced contrast of CT images ▪ Increased free radical generation via the Schottky barrier ▪ Exhibited intrinsic radiosensitizing ability	[193]

As described in Table 5, a variety of metal-based theranostics with different degrees of structural complexity and anticancer functionality has been tested. For instance, the simplest nanosystem included in the table (Ag NPs developed by Haque et al. [128]) required no anticancer drug, targeting moiety, or fluorescent molecule to work as an anticancer agent while also exhibiting NIR imaging potential. However, the encapsulation of various drugs (e.g., curcumin, melanin, doxorubicin, and methotrexate), the addition of different coatings (e.g., starch, calcium phosphate, and amorphous silica), and functionalization with certain biomolecules (e.g., folic acid, polyvinyl pyrrolidone, and split DNAzyme precursor) were demonstrated to significantly boost antitumor activity, ensuring specific targeting ability and controlled drug-release. Furthermore, these complex nanosystems allowed the simultaneous use of several approaches for fighting tumor growth. Specifically, there have been reported synergistic combinations integrating two or more of the following therapeutic and imaging modalities: chemotherapy, radiotherapy, PTT, PDT, magnetic hyperthermia, fluorescence imaging, MRI, NIR-based imaging, photoacoustic imaging, and CT imaging. Hence, metal-based theranostics have tremendous potential in developing highly performant chemotherapeutics, being expected to revolutionize cancer management in future years.

## 4. Conclusions

In summary, the development of metal nanoparticles is rapid and multidirectional, providing alternative treatment strategies and enhancing the outcomes of many cancer therapies. An increasing number of in vitro and in vivo studies has emerged in the specialty literature, showing promising results in the treatment of various cancers when using metal-based NPs with intrinsic anticancer properties or metallic nanoplatform in combinatorial approaches with other therapeutic options. In particular, the use of controlled-release systems triggered by pH, temperature, electromagnetic waves, light, and enzymes brings critical precision to the delivery of chemotherapeutics, improving accumulation in tumor tissues and strengthening therapeutic results.

However, most metal-based formulations have not yet been translated to clinical settings, mainly due to toxicity concerns. The abilities of metallic NPs to generate ROS, induce oxidative stress, disturb cytoskeleton integrity, and damage DNA, which are the main reasons for choosing them as cytotoxic agents, also represent obstacles in their approval. Thus, it is important that future tests also focus on enhancing the biocompatibility of these platforms in healthy tissues, gathering more evidence on their safety profiles and long-term outcomes. Another significant concern related to metallic nanoparticles is their stability in biological media, which must be carefully tailored by using surface functionalization with various organic molecules, macromolecules, or noble metal coatings.

In conclusion, metal-based nanoconstructs hold great promise for developing more performant anticancer therapies, and they deserve further special interdisciplinary research efforts towards overcoming current limitations.

## Figures and Tables

**Table 1:** Half-maximal inhibitory concentrations (IC50) of Ag NPs against various human cancer cell lines.

Cancer Type	Cell Line	IC50 (μg/mL)	Ref.
Liver cancer	HepG2	48	[52]
	HepG2	75	[53]
Breast cancer	MCF-7	20	[55]
	MCF-7	0.65	[56]
	AU565	0.25	[56]
	T47D	5	[57]
Ovarian cancer	PA-1	30	[58]
	A2780	7	[59]
	A2780Cis	14.04	[59]
Prostate cancer	PC-3	56.27 ± 1.17	[60]
Colon cancer	HCT-116	50	[30]
	HCT-116	1.152	[66]
	HT29	4.88	[63]
Lung cancer	A549	28	[58]
	A549	11.28 ± 1.28	[60]
Bone cancer	MG-63	0.665	[69]

**Table 3:** Examples of metal-based nanomaterials for cancer vaccine delivery.

Material	Morphology	Immunogen	Results	Ref.
Aluminum hydroxide	Nanospheres (modified with polyethylenimine)	Ovalbumin	▪ Easily internalized into DCs, ensuring antigen release into their cytoplasm ▪ Significantly inhibited tumor growth ▪ Considerably increased cytokine IL-12 secretion and expression of surface molecules CD80 and CD86 ▪ Promoted the activation of tumor-associated T cells	[148]
Iron oxide	Nanospheres	Ovalbumin	▪ Considerably promoted activation of immune cells ▪ Significantly increased cytokine production ▪ Induced potent humoral and cellular immune responses	[149]
Iron oxide	Nanospheres (coated with a lipid bilayer)	Endogenous tumor antigens (ETAs)	▪ Able to capture ETAs from tumors and transport them to lymph nodes ▪ In combination with anti-PD-L1 checkpoint blockade could eliminate primary tumors, suppress distant tumors, inhibit metastasis, and prolong the survival of model animals	[150]
Zinc oxide	Mesoporous nanocapsules	Ovalbumin	▪ Enhanced expression of antigen-specific T-cells ▪ Induced IFN-γ producing effector CD4+ and CD8+ T-cells ▪ Increased antigen-specific IgG levels	[151]
Zinc oxide	Radially grown nanowires on poly-L-lactide microfibers	Carcinoembryonic antigen	▪ Mild cellular toxicity ▪ Effective delivery to DCs, stimulating them to express inflammatory cytokines and activation surface markers ▪ Induced tumor antigen-specific cellular immunity ▪ Significantly inhibited tumor growth ▪ Reduced immune suppressive T_Reg_ cells ▪ Enhanced the infiltration of T cells into tumor tissues	[152]
Magnesium-aluminum-layered double hydroxide	Nanospheres	Tyrosinase-related protein 2	▪ Induced strong cytotoxic T-lymphocyte responses ▪ Significantly inhibited melanoma tumor growth ▪ The NPs allow loading of multi-antigens and immune stimulants, being promising for developing personalized therapeutic cancer vaccines	[153]
Calcium phosphate	Nanospheres (functionalized with lipids)	p-AH1-A5 peptide antigen	▪ Reduced primary colon cancer growth rate ▪ Arrested liver metastasis ▪ Boosted the adaptive CD8+ T-cell population, without inciting increased populations of immune suppressive cell types (e.g., T-regulatory cells and myeloid derived suppressor cells)	[154]

## Data Availability

Not applicable.
